# The Performance of the Endurant Endoprosthesis in an Infrarenal Aortic Aneurysm with a Wide or Conical-Shaped Infrarenal Neck Anatomy

**DOI:** 10.3390/jcm14124133

**Published:** 2025-06-11

**Authors:** Maaike Plug, Suzanne Holewijn, Armelle Meershoek, Daphne van der Veen, Michel M. P. J. Reijnen

**Affiliations:** 1 Department of Surgery, Rijnstate Hospital, Wagnerlaan 55, 6815 AD Arnhem, The Netherlands; mplug@rijnstate.nl (M.P.); ameershoek@rijnstate.nl (A.M.);; 2 Multi-Modality Medical Imaging Group, TechMed Centre, University of Twente, 7522 NB Enschede, The Netherlands

**Keywords:** abdominal aortic aneurysm, endovascular abdominal aneurysm repair, EVAR, wide neck, conical neck, hostile neck

## Abstract

**Background/Objectives:** Wide and conical-shaped infrarenal necks are risk factors for neck-related complications after Endovascular Aorta Aneurysm Repair (EVAR). The aim of this study is to investigate the performance of the Endurant endoprosthesis in wide/conical-shaped aortic neck anatomies compared to its performance in a normal infrarenal neck (reference group). **Methods**: A single-center, retrospective observational cohort study was performed, including consecutive subjects with an infrarenal abdominal aortic aneurysm, treated electively with an Endurant endoprosthesis. The primary endpoint was the freedom from aneurysm-related reinterventions through 1 year. Secondary endpoints included proximal fixation failure, type IA endoleak, stent migration, aneurysm sac remodeling, aneurysm-related mortality, freedom from reinterventions throughout available follow-up, and rupture. **Results**: A total of 268 patients were included, with a mean age of 73.3 years, and 85.1% were male. Freedom from aneurysm-related reinterventions was significantly lower in the wide-neck group (60.0%) compared to the reference group (81.1%; *p* = 0.018) but not for the conical-neck group (70.3%; *p* = 0.286). Median time to first reintervention was 1.7 (IQR 0.8; 4.4 years) in the reference group, 2.9 years (IQR 0.3; 5.0 years) in the wide-neck group (*p* = 0.547) and 3.8 years (IQR 0.4; 6.5) in the conical-neck group (*p* = 0.123). The proximal fixation failure rate was 7.4% in the wide-neck group compared to 3.3% in the reference group (*p* = 0.155) and 1.7% in the conical-neck group (*p* = 0.525). The type IA endoleak rate was 4.9% in the wide-neck group versus 3.3% in the reference group (*p* = 0.250). **Conclusions**: In the group with wide necks, reintervention-free survival was lower compared to the reference group, which seems to be driven by proximal fixation failure.

## 1. Introduction

Hostile infrarenal neck anatomies represent a common cause for the exclusion of patients for endovascular aneurysm repair (EVAR). It increases the risk of complications such as endoleaks, stent migration, aneurysm rupture, and mortality [1,2,3,4]. Consequently, the incidence of reinterventions is higher in patients with a hostile neck anatomy, which subsequently increases the costs [1,2,3]. Definitions of how a hostile neck is defined vary in the literature. In this study, we focus on two hostile neck anatomies: the wide and the conical-shaped neck. Wide neck and conical neck are two distinct hostile neck parameters that may co-occur but are not mutually inclusive. The definition of these hostile neck anatomies varies in studies, making the literature not uniform. The most commonly used definition of the wide neck is an aneurysm neck of >28 mm, which was also adapted by the Society for Vascular Surgery [1,5]. Several definitions of a conical neck have been described, such as a diameter increase of >10% or a diameter increase of 2 or 3 mm, both measurements within the first 10 or 15 mm from the most caudal renal artery [1,6,7,8]. For this study, all definitions were assessed. The Endurant Endoprosthesis (Medtronic, Santa Rosa, CA, USA) was introduced in 2008 and was specially designed to overcome anatomical limitations, including a wide and conical neck anatomy. The suprarenal fixation and wire-formed M-shaped body, enhancing conformability, ensure an adequate proximal fixation and seal, which in turn may result in fewer complications such as endoleaks [3,4,9]. The aim of this study was to investigate the performance of the Endurant endoprosthesis in a wide or conical-shaped infrarenal neck anatomy compared to the performance in a ≤28 mm normal neck group.

## 2. Materials and Methods

This is a single-center, retrospective observational cohort study. Consecutive subjects with an infrarenal abdominal aortic aneurysm (AAA), who were treated with an Endurant endoprosthesis during the period of January 2011 up until January 2020, were included. Every subject matched the aneurysm criteria to receive elective EVAR; AAA’s are larger than 5.5 cm for men or 5.0 cm for women, and they are rapidly expanding. Subjects who underwent non-elective EVAR and/or repair for an inflammatory aneurysm were excluded. In addition, isolated iliac aneurysms and subjects without contrast-enhanced computerized tomography (CTA) in follow-up were excluded. All other subjects were included in the analysis. A CTA was performed for preoperative planning using a 64-slice CT with and without contrast during arterial and venous phases at a thickness of 1 mm.

The retrospective research of patient files is not in the scope of Dutch law for human research. The study was conducted in accordance with the Declaration of Helsinki and approved by the Institutional Review Board of Rijnstate (2021-1830; 23 March 2021). As a consequence, informed consent was not obtained from the patients. Electronic hospital records were checked to ensure patients had no objection to the use of data in scientific research. Patient data were coded and analyzed. Standard follow-up consists of a CTA, duplex ultrasound, and clinical consultation at 6 weeks, one year, and yearly thereafter.

The definitions used in this research were based upon the reporting standards according to the Society for Vascular Surgery, derived from Chaikof et al., and the 2018 practice guidelines on the care of patients with an abdominal aortic aneurysm by The Society for Vascular Surgery [1,5].

A wide neck was defined as a diameter > 28mm, according to the Delphi consensus study by Marone et al. [1].

Four definitions of the conical neck were investigated according to different studies; for this study, all definitions were evaluated [6,7,8]. These definitions include:

1. A diameter increase >10% compared with the immediate infrarenal diameter over the first 10 mm beyond the lowest main renal artery;
2. The neck dilated >2 mm within 10 mm of the most caudal renal artery;
3. The neck dilated >3 mm within 15 mm of the most caudal renal artery;
4. The diameter increased >10% compared with the immediate infrarenal neck diameter over the first 15 mm beyond the lowest main renal artery.

The reference group consisted of patients with a diameter ≤28 mm and a straight neck anatomy. Proximal fixation failure was defined as a composite of type IA endoleak and stent graft migration > 10 mm after EVAR during available follow-up [10]. The oversizing percentage was calculated as the prosthesis diameter minus neck diameter, divided by neck diameter × 100.

Sac remodeling was classified as growth if the aneurysm sac increased by more than 5 mm, stable if it changed by 5 mm or less, and shrinkage if the aneurysm sac decreased by more than 5 mm. Aneurysm-related mortality is a death related to an aneurysm, such as a rupture. Overall mortality was defined as the death of patients due to any cause, for example, pneumonia, trauma, or cardiac arrest.

All AAA-related measurements were independently performed by two researchers (M.P. and A.M.), using Philips IntelliSpace Cardiovascular 5.1 to analyze preoperative CTAs (Phillips, Best, The Netherlands). The measurements were performed on center lumen-line reconstructed imaging and consisted of four diameters of the aortic neck: immediately distal of the lowest renal artery, and 5 mm, 10 mm, and 15 mm below this point (if applicable). Furthermore, the length of the aortic neck (measured from the lowest renal artery up until the beginning of the aneurysm) and the β-angle (angle between the aneurysm neck and centerline of the aneurysm) of the neck were measured. Finally, in the case of discrepancies between the two researchers, the assessment was repeated to come to an agreement. The average of the four diameters of the aortic neck was calculated to classify the subjects into groups based on their neck characteristics, namely the straight and non-wide neck or wide neck. For the conical neck, an increase in the diameters was used, according to the definitions used, to determine conical necks. 

The primary outcome of this study was freedom from reinterventions. The freedom from reinterventions is measured in the percentage of patients that did not require AAA-related intervention at the end of the first year, measured in months, and until the first AAA-related reintervention until the latest follow-up available, measured in years.

The secondary outcomes included AAA-related death, endoleak, aneurysm rupture, stent migration, proximal fixation failure, and sac remodeling.

Continuous variables are presented as mean and standard deviation (SD) or median and interquartile range (IQR) if applicable. Normality was tested using Kolmogorov–Smirnov test. The groups of patients with a wide aortic neck and a conical aortic neck were both compared to the reference group. The groups were analyzed as the reference group, wide aortic neck group, and conical aortic neck group. Differences between the groups were analyzed using the chi-square for nominal variables and the *t*-test or Mann–Whitney U test for normally distributed or non-normally distributed continuous variables, respectively. For the categorical variables, linear-to-linear test results were used to interpret more than two categories. Freedom from reinterventions was analyzed using a Kaplan–Meier analysis, and differences between groups were tested using the log-rank test or the Breslow test if applicable. Cox regression analyses were performed to investigate predictors for reinterventions for all subgroups. Statistical analysis was performed using IBM SPSS Statistics (SPSS version 29.0 for Windows, IBM Corporation, Armonk, NY, USA). A two-sided *p*-value < 0.05 was considered significant.

## 3. Results

**Figure 1:**
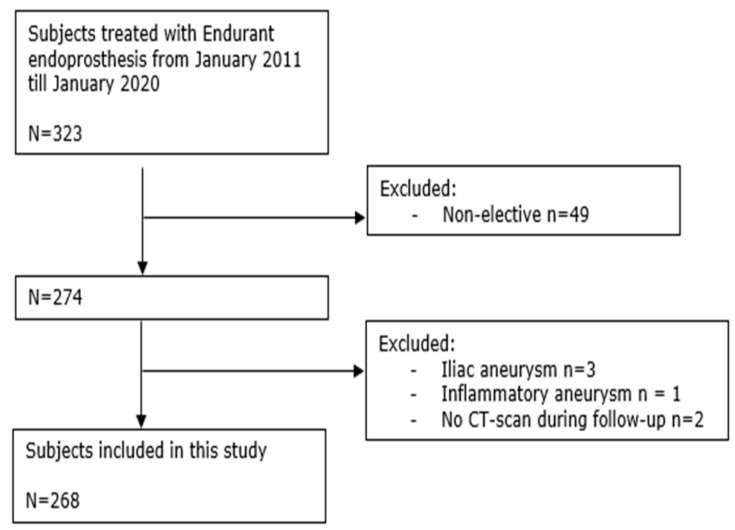
Flow chart with subjects treated with Endurant endoprosthesis with excluded subjects and a final number of subjects included in this study.

During the study period, 323 subjects were treated with Endurant endoprosthesis. Forty-nine subjects received an Endurant endograft in a non-elective setting (<24 h). Of the remaining 274 subjects, there were three that had an isolated iliac aneurysm, two that did not receive a follow-up CT scan, and one that had an inflammatory aneurysm (Figure 1). Overall, 268 subjects were enrolled, with a median follow-up of 45.4 months (IQR: 25.2; 86.6) in the whole population. The median follow-up of the three groups is 55.2 months for the reference group, 47.1 months for the wide neck, and 48.3 months for the conical-neck group. 

The population was divided into three groups: The reference group consisted of 153 patients; 81 patients presented with a wide infrarenal aortic neck, and 60 patients with a conical-shaped infrarenal aortic neck. Twenty-six subjects presented with both a wide and a conical neck and were therefore included in both study groups to account for this overlap. The various definitions of conical neck resulted in the identification of the exact same patient cohort; therefore, the impact of these differing definitions was not analyzed separately. Baseline and anatomical characteristics are presented in Table 1 and Table 2, respectively. In general, most of the subjects were male, over 70 years of age, used anticoagulant medication, and were on statin therapy. The majority of aneurysms were fusiform, with a median maximum diameter of 59.0 mm. Patients with either a wide or a conical neck had a significantly larger neck diameter when compared to the reference group (*p* < 0.001). There were no other statistical significances between groups regarding baseline and aneurysm characteristics besides fewer fusiform-shaped aneurysms in the reference group compared to the wide neck group (*p* = 0.036). The median infrarenal neck length was more than 20 mm in all three groups. The number of patients that had a neck length of less than 10 mm was one in all three groups. A total of 47 patients had a neck angulation of more than 60 degrees, including 28 patients in the reference group, 11 patients in the wide group and 8 patients in the conical group. 

**Figure 2:**
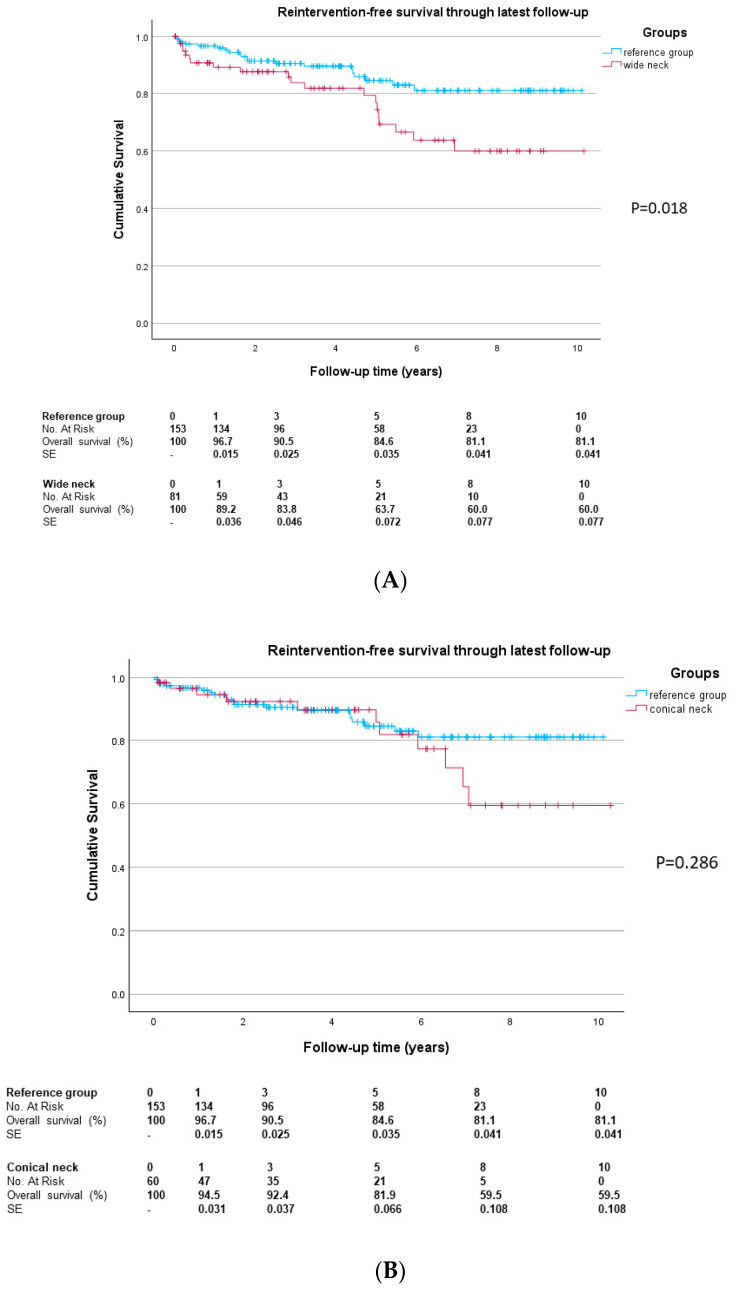
Freedom from aneurysm-related reinterventions during follow-up: (**A**) for the reference and wide neck groups, and (**B**) for the reference and conical-neck groups. No.—number; SE—standard error.

In the reference group, the freedom from aneurysm-related reinterventions (Figure 2) through one year was 95.6%, versus 88.5% in the wide neck group (*p* = 0.055) and 93.8% in the conical-neck group (*p* = 0.696). The difference between the wide and conical-neck groups was not significant (*p* = 0.281). The freedom from aneurysm-related reintervention rate during the entire follow-up was 81.1% in the reference group, 60.0% in the wide neck group (*p* = 0.018) and 59.5% in the conical-neck group (*p* = 0.286). The median time to first reintervention in the entire cohort was 1.8 years (IQR 0.4; 4.9 years). For the reference group, the median time to first reintervention was 1.7 years (IQR 0.8; 4.4 years). Compared to the reference group, the wide neck group showed a time to first reintervention of 2.9 years (IQR 0.3; 5.0 years; *p* = 0.547) and the conical-neck group 3.8 years (IQR 0.4; 6.5, *p* = 0.123). No significant difference in time to first reintervention was observed between the wide and conical-neck groups (*p* = 0.549). 

**Figure 3:**
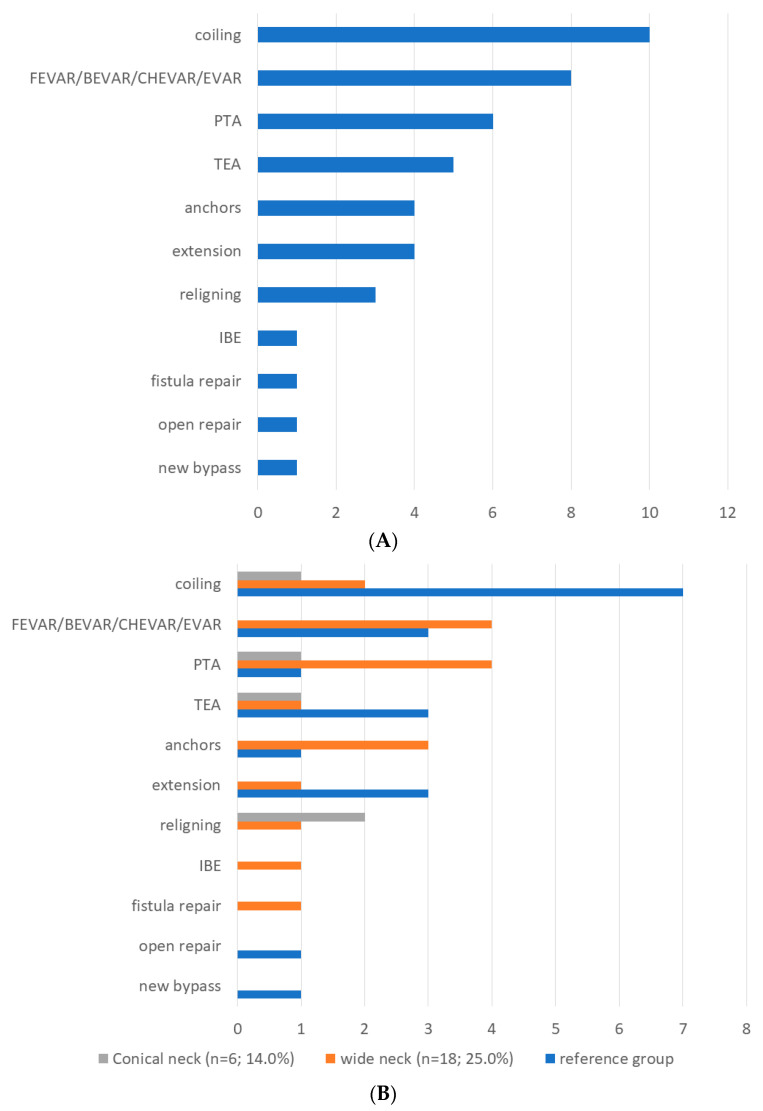
Overview of the first reinterventions performed overall (**A**) and by subgroup (**B**) during follow-up. PTA—Percutaneous transluminal angioplasty, FEVAR—Fenestrated endovascular aneurysm repair, BEVAR—Branched endovascular aneurysm repair, CHEVAR—Chimney endovascular aneurysm repair, EVAR—Endovascular aneurysm repair, TEA—Thromboendarterectomy, IBE—iliac branch endoprosthesis.

Overall, 44 patients needed AAA-related reinterventions during follow-up; 22 had one, 13 had two, 6 had three and 2 patients had four reinterventions. In Figure 3, the type of first reintervention is depicted for the total group (A) and by subgroup (B). In the reference group, 20 patients had a reintervention; in the wide-neck group, 18, and in the conical-neck group, 6 patients had reinterventions during follow-up. Of the patients who needed four reinterventions, one was in the reference group and one in the wide neck group.

Univariate Cox Regression analyses on reintervention-free survival showed Hazard Ratios (HR) of 2.08 (95% CI 1.12; 3.86) for the wide-neck group compared to the reference group, 1.49 (95% CI 0.71; 3.11) for conical neck compared to the reference group, and 0.49 (95% CI 0.16; 1.49) for conical necks compared to wide necks. In multivariate Cox Regression analyses analyzing all groups together, only those free from renal disease (HR 0.33, 95% CI 0.11; 0.96) and having an endoleak during follow-up (HR 7.8, 95% CI 3.44; 18.00) showed significant impact on reintervention-free survival. The covariates in the multivariate model included group, age, gender, baseline AAA diameter, aneurysm type, baseline infrarenal neck length, beta angle, ASA group, hypertension, diabetes, hyperlipidemia, renal disease at baseline, current smoking, history of cardiac disease, procedure time, endoleak during follow-up, and percentage oversizing. Subgroup analyses were also performed. In the reference group, an endoleak during follow-up was a significant predictor of reinterventions (HR 24.48, 95% CI 520; 115.18), and percentage oversizing was a significant predictor of reinterventions (HR 1.07, 95% CI 1.01; 1.13). In the cohort of wide-neck patients, baseline AAA diameter (HR 1.10, 95% CI 1.00; 1.21), infrarenal aortic neck length (HR 1.12, 95% CI 1.02; 1.24), beta angle (HR 0.928, 95% CI 0.86; 0.99), and hypertension at baseline (HR 0.15, 95% CI 0.00; 0.24) were associated with reinterventions. In the group of conical necks, no significant predictors for reinterventions were found. 

The secondary endpoints are shown in Table 3. The percentage oversizing was highest in the conical-neck group (18.5%, *p* = 0.317 compared to the reference group (16.7%)). Percentage oversizing was lowest in the wide-neck group (12.3%, *p* = 0.010 compared to the reference group). The proximal fixation failure rate was more than 2-fold higher in the wide-neck group when compared to the reference group (7.4% vs. 3.3%, *p* = 0.155) and lower in the conical-neck group (1.7%, *p* = 0.525). Similarly, the type IA endoleak rate was slightly higher in the wide-neck group when compared to the reference group (4.9% vs. 3.3%, *p* = 0.250) and lower in the conical-neck group (1.7%, *p* = 0.725). Aneurysm rupture did not occur in the reference group and conical-neck group, but two patients with wide neck anatomy experienced a rupture at 2 and 34 months postoperatively, respectively (*p* = 0.051). The aneurysm-related mortality was 2.0% in the reference group, 3.7% in the wide-neck group (*p* = 0.770), and 3.3% in the conical-neck group (*p* = 0.689). 

**Figure 4:**
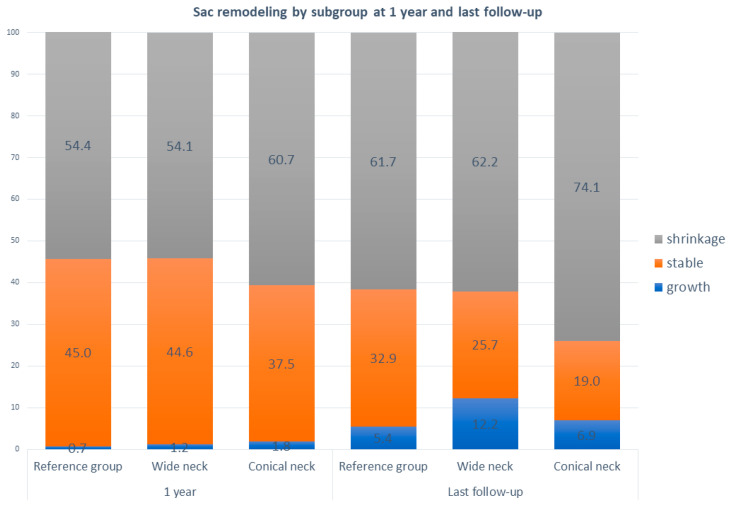
Bar chart sac remodeling at one year and last follow-up after EVAR (%). Growth is defined as >5 mm growth during follow-up and shrinkage as <5 mm during follow-up. All other measurements were defined as stable sac.

Aneurysm sac remodeling is shown in Figure 4. Within all groups, most aneurysms shrank over time. No significant differences in sac remodeling were found at 1 year or the latest follow-up compared to the reference group. At 1 year follow-up, the percentage of growth was low (0.7–1.8%) in all groups. At the latest follow-up, the percentage of growth varies between 4.1% and 7.0%. No difference in sac remodeling at 1 year was found between wide and conical neck patients (*p* = 0.287), while at the last follow-up, the difference between groups was significant in favor of the conical-neck group; *p* = 0.028. 

## 4. Discussion

Our results show high percentages of freedom from interventions during follow-up for patients with an AAA treated with the Endurant endograft. However, patients with a wide infrarenal neck need more reinterventions during follow-up compared to those with conical necks and the reference group. Although wide-neck and conical neck represent separate hostile neck parameters, they are not mutually exclusive. As a result, overlap between the groups may occur, with 26 patients exhibiting both features in our study. The sample size in this subgroup is considered too small to draw definitive conclusions. Patients with a wide infrarenal neck had lower freedom from aneurysm-related reinterventions at 1-year follow-up (respectively 88.5%) compared to the reference group (95.6%). They also have a trend towards more proximal fixation failures and type Ia endoleak. Both neck diameter and excessive oversizing have been identified as risk factors for neck dilatation after EVAR, which is likely to be an important factor in proximal fixation failures [10,11]. Interestingly, in the current study, the amount of oversizing was lower in the wide-neck cohort compared to the reference group. 

Our findings align with the outcomes reported in the systematic review by Kouvelos et al. [12]. Their analysis of six observational studies showed significantly lower freedom from aneurysm-related reinterventions among the wide-neck aneurysm group after EVAR. Furthermore, they demonstrated a higher percentage of type Ia endoleak, migration, and aneurysm rupture. In that study, there was a variety in the definition of a wide neck. Jim et al. found more early adverse events and more migration at long-term follow-up in patients with a wide neck [11]. 

Conicity was the second hostile neck parameter that was studied. In this group, there was a trend towards more adverse events, but surprisingly, it was to a lesser extent when compared to the large necks. The reasons for this are unclear, but the smaller diameter of the neck just below the renal arteries might positively influence the fixation and seal in this area. In this study, various definitions of conicity were tested without impacting the groups. This indicates that the studies using different definitions could be comparable, but there is a clear need for standardization. To exclude heterogeneity in future research, the use of one single definition would be advocated.

Our results cannot be generalized to other devices with a different design. The M-shaped proximal ring stent of the Endurant ensures a good conformability that might positively impact the outcomes in the studied hostile neck parameters, particularly the conical-neck group. McFarland et al. also studied the differences in the performance of 7 different endografts, including the Endurant endoprosthesis, focusing on the failure of proximal fixation in subjects with a wide-neck anatomy. They showed only a significant difference in freedom from proximal fixation failure in subjects with a wide neck treated with the Zenith endograft. Moreover, they reported an increased risk of proximal fixation failure when Talent endografts are used in the wide-neck group [10].

Although we show an increased incidence of type Ia endoleak, we did not observe differences in the sac remodeling between the three subgroups. Sac remodeling is becoming increasingly important in the follow-up patients treated with EVAR, as it is related to the correlation of sac shrinkage at one year with mortality and reinterventions. The incidence of sac growth and shrinkage was low and in line with the data published in the literature. 

As an alternative for increasing the sealing length in the hostile neck, additional fixation using EndoAnchors, so-called EndoSuture aneurysm repair (ESAR), might also improve outcomes. In the PERU registry, it was shown that adjunct EndoAnchor usage at EVAR achieves high rates of freedom from type Ia endoleak at mid-term, including in a high number of patients with hostile neck anatomy, with positive trends in sac-size evolution [13]. These data confirmed the outcomes of the ANCHOR registry that showed that the primary use of EndoAnchors in patients with a hostile neck anatomy is related to a low incidence of type Ia endoleaks [14]. The disadvantage of these studies is the lack of heterogeneity. A variety of hostile neck parameters were present, and consequently, it remained unclear which hostile neck parameter does benefit from adjunctive treatment. The currently enrolling HERCULES trial, randomizing patients with a wide infrarenal neck between EVAR and ESAR, will provide a more solid answer.

There are limitations to this study. First, only one single endograft device was studied, and consequently, results cannot be generalized. Second, this was a single-center study with a relatively small sample size, so this limits external validation and might have introduced selection and information bias. Furthermore, two researchers measured the diameter of the aortic neck and the angle of the aneurysm, potentially inducing an enrollment bias. To minimize bias, it would be optimal to develop an artificial intelligence program to assess the shape and diameter of the infrarenal neck. In accord with the conical neck, it is also not clear how to accurately measure the neck diameter of the infrarenal neck.

In this current study, we measured four diameters of the aortic neck, including the immediate distal of the lowest renal artery and 5 mm, 10 mm, and 15 mm below this point. We divided the subjects into groups based on the mean diameter of these four measurements. Lastly, neck features such as atherosclerotic debris, calcification, and thrombus were not measured. These factors may have negative effects on the clamp site of the endoprosthesis and thus might influence proximal fixation failure rates. In a future study with larger sample sizes, these factors should be included. Implications for clinical practice cannot be drawn based on this study. More prospective and comparative studies investigating this should be performed. The ongoing Socrates trial (NCT04503395) and the HERCULES trial (NCT05484115) should reveal more implications for clinical practice in patients with a challenging infrarenal neck anatomy.

## 5. Conclusions

In conclusion, we have demonstrated that the usage of the Endurant endoprosthesis is related to acceptable results in patients with an aneurysm with wide and/or conical neck, but more complications seem to occur, particularly in the wide-neck cohort and that more reinterventions are needed, although the differences did not reach statistical significance. Therefore, this study does not provide enough evidence to fully prove our hypothesis. This indicates that close surveillance is indicated in these subgroups. Uniformity in definitions of both hostile neck anatomies is strongly recommended.

## Figures and Tables

**Table 1:** Baseline patient characteristics by study group.

	Reference Group	Wide Neck	*p*	Conical Neck	*p*
N	153	81		60	
Male (%)	127 (83.0)	73 (90.1)	0.142	48 (80.0)	0.606
Age, mean (SD)	73.0 (7.9)	74.3 (8.8)	0.252	73.7 (7.6)	0.541
ASA, n (%) 1 2 3 4	1 (0.7) 67 (43.8) 66 (43.1) 19 (12.4)	0 (0.0) 34 (42.0) 37 (45.7) 10 (12.3)	0.747	0 (0.0) 23 (38.3) 32 (53.3) 5 (8.3)	0.794
Diabetes mellitus, n (%)	25 (16.3)	20 (24.7)	0.123	10 (16.7)	0.954
Current smoking, n (%)	59 (38.6)	32 (39.5)	0.888	22 (36.7)	0.798
Anti-coagulation, n (%)	153 (100.0)	81 (100.0)	1.000	60 (100.0)	1.000
Antiplatelet therapy n (%)	137 (89.5)	71 (87.7)	0.662	54 (90.0)	0.921
Vitamin K-antagonist n (%)	26 (17.0)	18 (22.2)	0.330	11 (18.3)	0.816
Dual therapy n (%)	31 (20.3)	18 (22.2)	0.726	16 (26.7)	0.311
Anti-hypertensive, n (%)	140 (91.5)	70 (86.4)	0.223	51 (85.0)	0.161
Statin, n (%)	122 (79.7)	72 (88.9)	0.135	53 (88.3)	0.219

Significance (*p*) is calculated between the wide neck and reference group or conical neck and reference group (*p* < 0.05). ASA denotes the American Society of Anesthesiologists physical classification system. Subjects with a wide and conical neck also add up to the wide-neck and conical-neck groups.

**Table 2:** Aneurysm characteristics by study group.

	Reference Group	Wide Neck	*p*	Conical Neck	*p*
N	153	81		60	
Type of aneurysm, n (%) Saccular Fusiform	8 (5.2) 145 (94.8)	0 (0.0) 81 (100.0)	0.036	1 (1.7) 59 (98.3)	0.245
Infrarenal aortic neck length, median (IQR)	23.5 (16.0; 37.0)	27.0 (19.0; 38.0)	0.232	21.0 (15.0; 35.0)	0.903
Infrarenal aortic neck diameter, median (IQR)	24.0 (22.0; 26.0)	30.5 (29.0; 32.5)	<0.001	27.8 (23.0; 30.5)	<0.001
Maximal diameter AAA in mm, median (IQR)	58.0 (54.0; 64.0)	59.0 (56.0; 67.0)	0.138	59.0 (54.0; 66.5)	0.175
Angle AAA (o), median (IQR)	39.6 (20.7; 51.3)	38.7 (27.4; 55.0)	0.778	36.8 (24.3; 52.8)	0.999
Angle AAA > 60°, yes (n)	28	11	-	8	-
Common iliac artery diameter, median (IQR) Left Right	14.0 (12.0; 18.0) 15.0 (13.0; 20.0)	16.0 (14.0; 20.0) 15.0 (14.0; 20.0)	0.131 0.390	16.0 (13.0; 18.0) 15.5 (14.0; 20.0)	0.319 0.389
Common iliac artery aneurysm (yes), n (%) Left Right Both	4 (2.6) 12 (7.8) 11 (7.2)	2 (2.5) 4 (4.9) 5 (6.2)	0.459	3 (5.0) 6 (10.0) 5 (8.3)	0.475
Instructions for use, N (%)	104 (68.0)	44 (61.1)		38 (63.3)	

Significance (*p*) is calculated between the wide neck and reference group or conical neck and reference group (*p* < 0.05). Subjects with a wide and conical neck also add up to the wide-neck and conical-neck groups. The number of endografts treated according to their instruction for use (IFU) is depicted in percentages in the various groups.

**Table 3:** Secondary endpoints by study group.

	Reference Group	Wide Neck	*p*	Conical Neck	*p*
N	153	81		60	
Percentage oversizing	16.7 (9.8; 21.7)	12.3 (9.4; 19.5)	0.010	18.5 (13.7; 21.9)	0.317
Endoleaks, n (%) (first) Type 1A Type 1b Type 2 Type 3 Type unknown	33 (21.6) 5 (3.3) 0 (0.0) 20 (13.1) 0 (0.0) 8 (5.2)	24 (29.6) 4 (4.9) 4 (4.9) 11 (13.6) 0 (0.0) 5 (6.2)	0.172 0.250	12 (22.2) 1 (1.7) 2 (3.3) 8 (13.3) 1 (1.7) 3 (5.0)	0.590 0.725
Aneurysm rupture, n (%)	0 (0.0)	2 (2.5)	0.051	0 (0.0)	1.000
Stent migration, n (%)	1 (0.7)	2 (3.0)	0.352	0 (0.0)	0.681
Proximal fixation failure, n (%)	5 (3.3)	6 (7.4)	0.155	1 (1.7)	0.525
Endoleak 1a Both endoleak 1a and migration	4 (2.6) 1 (0.7)	4 (4.9) 2 (2.5)	0.576	1 (1.7) 0 (0.0)	0.833
AAA-related reintervention, n (%)	20 (13.1)	20 (24.7)	0.025	11 (18.3)	0.327
Amount of reinterventions, number of patients 1 reintervention 2 reinterventions 3 reinterventions 4 reinterventions	9 6 4 1	11 5 2 1	0.599 0.850 0.427 0.000	3 2 0 0	0.600 0.109 - -
AAA-related mortality, n (%)	3 (2.0)	2 (3.7)	0.770	2 (3.3)	0.689
Overall mortality, n (%)	77 (50.3)	61 (75.3)	<0.001	36 (60.0)	0.203

AAA—abdominal aortic aneurysm. Significance (*p*) is calculated between the wide-neck and reference group or conical neck and reference group (*p* < 0.05). Subjects with a wide and conical neck also add up to the wide-neck and conical-neck groups.

## Data Availability

The raw data supporting the conclusions of this article will be made available by the authors upon request and can only be shared anonymously. This restriction is due to hospital policies, which prohibit data sharing without clearly defined purposes. Data transfer agreements must be in place before data can be shared.

## Abbreviations

The following abbreviations are used in this manuscript:

AAA	Abdominal aortic aneurysm
ASA	American Society of Anesthesiologists physical classification system
BEVAR	Branched endovascular aneurysm repair
CHEVAR	Chimney endovascular aneurysm repair
CI	Confidence interval
CTA	Computed tomographic angiography
ESAR	EndoSuture aneurysm repair
EVAR	Endovascular aneurysm repair
FEVAR	Fenestrated endovascular aneurysm repair
HR	Hazard ratio
IBE	Iliac branch endoprosthesis
IQR	Interquartile range
No.	Number
P	Significance
PTA	Percutaneous transluminal angioplasty
SD	Standard deviation
SE	Standard error
TEA	Thromboendarterectomy
